# Effect of Neem (*Azadirachta indica*) on the Survival of *Escherichia coli* O157:H7 in Dairy Manure

**DOI:** 10.3390/ijerph120707794

**Published:** 2015-07-10

**Authors:** Subbarao V. Ravva, Anna Korn

**Affiliations:** 1 Produce Safety and Microbiology Research Unit, United States Department of Agriculture, Agricultural Research Service, Western Regional Research Center, Albany, CA 94710, USA; 2 Foodborne Toxin Detection and Prevention Research Unit, United States Department of Agriculture, Agricultural Research Service, Western Regional Research Center, Albany, CA 94710, USA; E-Mail: annamkorn@gmail.com

**Keywords:** neem, *Azadirachta indica*, *E. coli* O157:H7, bioscreen, survival, dairy manure, neem extracts, azadirachtin

## Abstract

*Escherichia coli* O157:H7 (EcO157) shed in cattle manure can survive for extended periods of time and intervention strategies to control this pathogen at the source are critical as produce crops are often grown in proximity to animal raising operations. This study evaluated whether neem (*Azadirachta indica*), known for its antimicrobial and insecticidal properties, can be used to amend manure to control EcO157. The influence of neem materials (leaf, bark, and oil) on the survival of an apple juice outbreak strain of EcO157 in dairy manure was monitored. Neem leaf and bark supplements eliminated the pathogen in less than 10 d with a D-value (days for 90% elimination) of 1.3 d. In contrast, nearly 4 log CFU EcO157/g remained after 10 d in neem-free manure control. The ethyl acetate extractable fraction of neem leaves was inhibitory to the growth of EcO157 in LB broth. Azadirachtin, a neem product with insect antifeedant properties, failed to inhibit EcO157. Application of inexpensive neem supplements to control pathogens in manure and possibly in produce fields may be an option for controlling the transfer of foodborne pathogens from farm to fork.

## 1. Introduction

Concentrated animal feeding operations generate large amounts of manure waste [1], thus raising concerns about foodborne pathogen contamination of fruit and vegetable crops grown in the vicinity. A mid-sized dairy produces more than 12 million kilograms of manure per year [2] and the manure is usually stored on-site. This further increases the risk of pathogen contamination of produce grown nearby. Ruminants are primary reservoirs of many enteric pathogens including *Escherichia coli* O157:H7 (EcO157) [3,4]. EcO157 can cause life-threatening hemorrhagic colitis and in very severe cases causes hemolytic uremic syndrome [5]. Nineteen percent of all EcO157-associated outbreaks during 1998 to 2007 were due to the consumption of contaminated produce [6]. Pathogens attached to contaminated “ready to eat” produce are difficult to remove [7]. Therefore, prevention of pre-harvest contamination is critical. Thus, designing effective and inexpensive on-farm control strategies is essential.

Neem (*Azardirachta indica*) is a traditional and naturally available medicinal plant in India, South Africa, and Southeast Asia [8]. Almost every part of the neem tree has beneficial properties. Neem trees are grown extensively for their shade in India, for firewood in Ghana, and for reforestation in West Africa [9]. For centuries, neem twigs were used as teeth cleaning devices [9] as they are effective as antiplaque and anti-gingivitis agents [10] and thus some commercial herbal toothpastes contain neem as an active ingredient [11]. Water extracts of neem twigs inhibited growth of dental caries organisms *Streptococcus mutans, S. salivarius, S. mitis*, and *S. sanguis* [12]. Neem extracts have been reported to possess antibacterial, antifungal, antimalarial, and antiviral properties [10,13]. Neem leaves are used in India for curing diarrhea and cholera [14]. In addition, neem oil and leaves are used in popular medicine as antiparasitic, anti-inflammatory, antiulcer, antihyperglycemic, anticarcinogenic, and immunomodulatory agents [15,16]. Neem materials also affect more than 200 insect species as well as some mites and nematodes [9]. For example, an active ingredient from neem, azadirachtin, disrupts the metamorphosis of insect larvae and is thus used as a feeding deterrent [9]. Neem supplements and extracts inhibit many bacterial pathogens. Chloroform extracts of neem inhibited the growth of *Listeria monocytogenes* while ethanolic extracts showed higher inhibition for *Staphylococcus aureus* [17]. A water-soluble glycolipid, sulfonoquinovosyldiacylglyceride, isolated from the leaves of neem showed inhibitory activity against *Salmonella typhi*, *Shigella dysenteriae*, *E. coli*, and *Vibrio cholerae* [13]. Aquaneem, an emulsified product from neem kernels, inhibited pathogens of fish (*Aeromonas hydrophila, Pseudomonas fluorescens*, and *E. coli*) [18]. Extracts of neem cake, a waste byproduct of oil extraction, inhibited *Campylobacter jejuni* [19]. Extracts from neem leaves, seeds, and bark also act as nitrification inhibitors [20]. Thus, neem is proven to be effective against many bacterial pathogens including *E. coli*, but not against EcO157 according to an isolated study [17]. 

We evaluated the survival and fate of an outbreak related strain of EcO157 in manure supplemented with neem materials (leaf, bark, and oil). Manure was from a medium-sized dairy in the central valley of California. The extractable fractions of neem leaves were also evaluated for their influence on the growth of EcO157 in 300-µL microcosms of nutrient medium. Microcosms were designed to determine the nature of extracts responsible for pathogen inhibition by neem.

## 2. Experimental Section

### 2.1. Neem Materials and Extracts

Neem oil, powdered leaf, and bark were obtained from Neem Tree Farms (Brandon, FL, USA). An aqueous extract of neem was prepared by extracting 50 g leaf powder with 200 mL RO pure deionized water. Extraction was carried out by shaking for 1 h on a gyratory shaker and the aqueous supernatant was separated by centrifugation at 10,000× *g* for 10 min. The aqueous fraction was concentrated to 40 mL by rotary flash evaporation at 40 °C. The residual leaf paste was extracted by mixing for 1 h with 250 mL of 1:1 ethanol-ethyl acetate on a gyratory shaker. The organic extract was concentrated by flash evaporation and reconstituted in 25 mL ethanol. This extract from hereon will be called “ethyl acetate extract”. Five milliliters of the ethanolic extract were evaporated to dryness and reconstituted in 50 mL ethyl acetate and extracted with 100 mL of 1% sodium bicarbonate to remove the acidic components. The bicarbonate-washed ethyl acetate fraction was concentrated to dryness, reconstituted in 5 mL ethanol, and used in assays to determine the influence of neem extracts on EcO157. Dilutions of both “ethyl acetate extracts” were made in ethanol.

### 2.2. Influence of Neem Materials on the Survival of EcO157 in Dairy Manure

Dairy manure used in this study was collected from a medium-sized dairy in Oakdale, CA, USA [21,22]. Survival of a green-fluorescent-protein (GFP) labeled EcO157 strain, MM123, was monitored in triplicate 10 g manure samples supplemented with 0%, 0.5%, and 5% levels (on a weight basis) of leaf, bark, or oil. MM123 is a spontaneous rifampicin- (100 µg/mL) and nalidixic acid- (50 µg/mL) resistant mutant of GFP-labeled apple juice outbreak strain RM2315 (plasmid-born GFP; wild type: FDA strain SEA13B88) [23,24]. Double antibiotic resistance aids in discriminating MM123 from native organisms in manure [22]. Manure mixed thoroughly with neem materials was spread evenly in the bottom of 300 mL Erlenmeyer flasks. The manure had a pH of 6.9 and was moist and fluffy. The manure was inoculated with 2 mL of MM123 in 0.01 M phosphate-buffered saline (PBS, pH 7.4) containing 8.3 × 10^8^ CFU and thoroughly mixed prior to incubation at 37 °C for 10 days. Overnight growth of MM123 in LB broth supplemented with 50 µg/mL kanamycin (to select for GFP) was centrifuged and resuspended in PBS prior to inoculations. GFP-labeled EcO157 cells were monitored at various intervals from 100 mg manure samples. One hundred-microliter portions of 10-fold serial dilutions of manure in PBS were plated on LB agar supplemented with 100 µg/mL rifampicin, 50 µg/mL nalidixic acid, and 50 µg/mL kanamycin and incubated overnight at 37 °C. The fluorescent colonies of MM123 were counted on a UV Transilluminator (Fotodyne, Hartland, WI). Days for one log reduction (D-value) of the pathogen in manure were calculated from linear regressions of log cell number decline over time. 

### 2.3. Growth of EcO157 with Neem Extracts

Growth of EcO157 strain MM149 in half-strength LB broth supplemented with aqueous or organic extracts of neem was monitored in a Bioscreen C microbial growth curve analysis system (Growth Curves USA, Piscataway, NJ). MM149 was previously isolated from dairy manure and was chosen for its superior survival characteristics in dairy wastewaters [21,22]. Wells of Bioscreen plate contained 270 µL of Murashige and Skoog basal salts (Fisher Scientific, Fairlawn, NJ) with 50% LB broth at pH 7.0, 10 µL of neem extract, and 20 µL of the inoculum. Overnight growth of MM149 resuspended in 0.01M PBS was adjusted to an optical density of 0.6 at 600 nm prior to inoculation. The inoculated plates were constantly shaken at medium speed during incubation at 37 °C, and growth was monitored at 1-h intervals by an onboard spectrophotometer equipped with a wide-band filter (420 to 580 nm). The effect of neem extracts at full, 1/10th, and 1/100th strengths were compared with treatments containing 1, 10, 100, and 1000 µg/mL of azadirachtin. Treatments were not replicated. Inoculated wells containing 10 µL of ethanol serve as controls for solvent effects on the growth of MM149. 

## 3. Results

### 3.1. Survival of EcO157 in Dairy Manure Supplemented with Neem Materials

**Figure 1:**
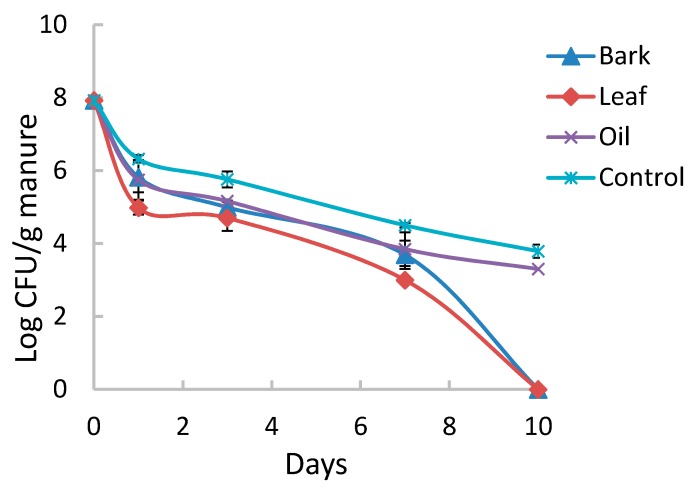
Growth of EcO157 in dairy manure supplemented with 5% neem materials.

Mixing neem leaf at a concentration of 5% in dairy manure resulted in a 3 log reduction in numbers of MM123 within one day of incubation (Figure 1) and both leaf and bark supplements at this level eliminated the organism in <10 d. However, a 1.5 log reduction in EcO157 populations occurred in a day after inoculation of neem-free manure controls. D-value calculated based on a 10-day incubation with bark or leaf was 1.3 ± 0.0 d as compared to 2.4 ± 0.1 d for neem-free controls. Neem materials at a lower concentration of 0.5% were not effective in inhibiting EcO157. Neem oil did not cause any significant decreases in numbers of EcO157 compared to an untreated control.

### 3.2. Growth of EcO157 with Neem Extracts

**Figure 2:**
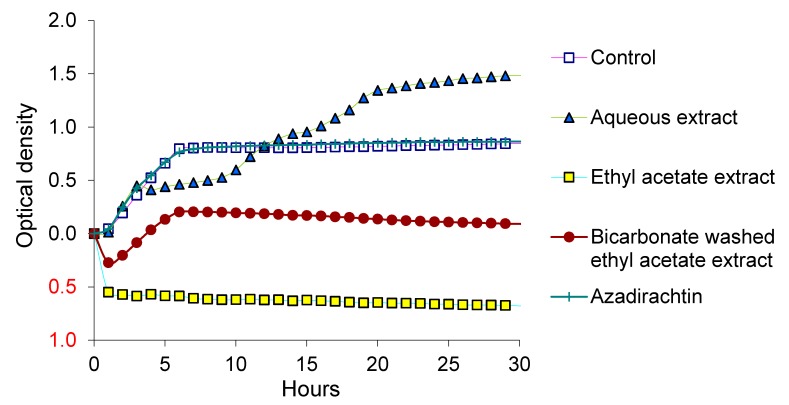
Growth of EcO157 in LB broth supplemented with full-strength aqueous or organic extracts of neem leaf. Optical densities at all sampling intervals were corrected for zero-time values. A control well contained 10 µL ethanol and 20 µL inoculum. Treatments of extracts at full strength (Table 1) were compared with azadirachtin at 1000 µg/mL.

**Figure 3:**
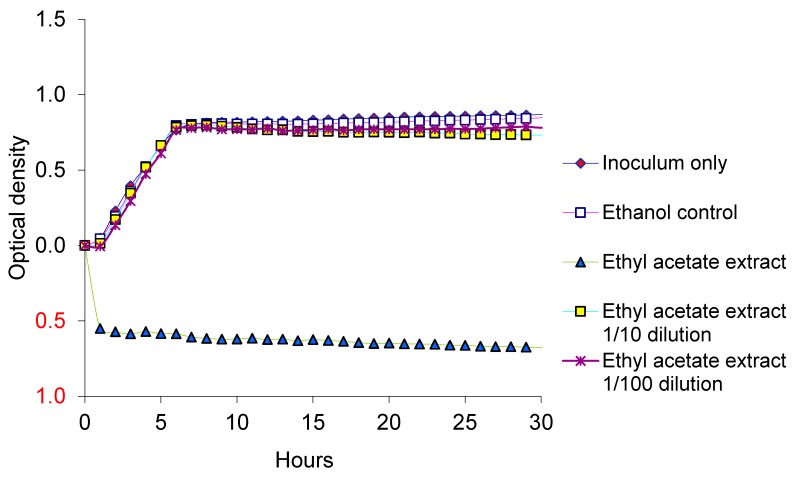
Growth of EcO157 in LB broth supplemented with ethyl acetate extracts of neem leaf. Optical densities at all sampling intervals were corrected for zero-time values.

The inhibitory effects of neem leaf and bark encouraged us to determine the extractable component of neem responsible for the inhibition of the pathogen. Since both leaf and bark behaved similarly in decreasing the populations of EcO157, aqueous and ethyl acetate extracts of leaves (Table 1) were evaluated for the inhibition of dairy manure isolate MM149. Ethyl acetate extract applied at full strength (Table 1) inhibited the growth of MM149, whereas aqueous extract supported its growth (Figure 2). Some inhibition was observed also with full-strength bicarbonate-washed ethyl acetate extract. Extracts tested at 1/10th and 1/100th dilutions behaved similarly as controls (Figure 3). Ethanol at 10 µL per well did not enhance or inhibit the growth. Growth was not inhibited by azadirachtin even at the highest concentration of 1000 µg/mL (Figure 2).

Optical densities measured at various sampling intervals were corrected for zero time values ranging from 0.05 to 0.6 for different treatments. The highest optical density at zero time was obtained with the full strength ethyl acetate extract. Some precipitation was noticed in this treatment but not at lower concentrations or with water or bicarbonate-washed ethyl acetate extracts. Clumping was not observed in any treatments. 

**Table 1:** Neem leaf extracts used in Bioscreen treatments.

Extract	Leaf Equivalents/Well ^a^, mg	Concentration/Well, %
Aqueous	12.5	4.2
Ethyl acetate	20	6.7
Bicarbonate washed ethyl acetate	20	6.7

Notes: ^a^ Each Bioscreen C well received 10 µL of the extract. This concentration was considered as full-strength and two ten-fold dilutions were also tested.

## 4. Discussion

EcO157 shed in feces of ruminants can survive for extended periods of time [25,26] and at times as long as 21 months in manure piles exposed to fluctuating environmental conditions [27]. In a recent study, we isolated a strain of EcO157 from dry produce field soil repeatedly during a 45-day period [26]. These observations indicate that some strains of EcO157 are very resilient and survive longer in austere environments and provide opportunities for the pathogen to be transported from farm to table. Intervention of pathogen transport from animal reservoirs to produce fields is essential. In this study, we explored the possibility of eliminating EcO157 from manure supplemented with neem materials known to have antimicrobial properties.

Neem leaf or bark eliminated the apple juice outbreak strain MM123 from dairy manure (Figure 1) in less than 10 days, whereas nearly 4 log CFU of the pathogen/g survived in manure without neem. A large effect of neem treatments did not become noticeable until after day 7 of the experiment. To our knowledge, this is the first report of neem supplements inhibiting the growth of EcO157 in manure. Although neem oil is used in popular medicines [16] and found to be bactericidal to *E. coli* [28], the oil is not effective against EcO157 in manure. However, extracts of waste byproducts (neem cake) after oil extraction inhibited *C. jejuni*, a foodborne pathogen associated with contaminated meat and poultry, in a broth model meat system [19]. Generic non-pathogenic *E. coli* were also inhibited in this model system, but it is not certain if the data can be extrapolated to pathogenic EcO157 or other shiga-toxigenic *E. coli*. Nonetheless, EcO157 in dairy manure was controlled by neem supplements.

Since neem supplements controlled EcO157 in manure, we explored the nature of the active ingredient. The solvent extractable organic fraction from neem leaves is effective in totally inhibiting the growth of EcO157 in LB broth. The removal of acidic organic components with bicarbonate also resulted in some inhibition of the pathogen. Thus, it appears that the inhibitory activity of neem leaves is inherent to the organic fraction extracted by ethyl acetate containing both basic and acidic components. In contrast, chloroform and ethanol extracts of neem were previously found to be not inhibitory to EcO157 but inhibited the growth of two other foodborne pathogens, *L. monocytogenes* and *S. aureus* [17]. Even though the organic extractable components are inhibitory to foodborne pathogens in this and a previous study, azadirachtin, a potent insect antifeedant [9] extracted from neem, was not found to be inhibitory to EcO157. In addition, we found that an aqueous extract of neem leaves enhanced the growth of EcO157 although water extracts of neem chewing sticks were found inhibitory to supra-gingival plaque organisms including generic *E. coli* [10]. However, in a different study, water extracts were not found to be inhibitory to multi-drug-resistant *E. coli* [29]. Thus, results from the current study are distinct in that pathogenic EcO157 are inhibited by supplements of neem leaf or bark and the active ingredient for inhibition is extractable by ethyl acetate. 

Foodborne pathogens in manure can be controlled with inexpensive treatments such as composting and/or supplementation of neem materials. Furthermore, pathogen transfer from point sources such as dairies and feedlots can be minimized by maintaining a safe setback distance for produce cultivation [30]. Produce fields can also be treated with neem supplements to control the pathogen transfer from soil to plants, although field tests are warranted to determine the efficacy of neem. Neem supplements could be a viable option for foodborne pathogen control wherever neem is grown. 

## 5. Conclusions

Foodborne pathogen contamination from “ready to eat” produce is difficult to remove. Treating with neem supplements could be an inexpensive way to prevent pre-harvest contamination via manure from nearby animal raising operations. Supplementation of neem leaf and bark to manure resulted in elimination of pathogenic EcO157 in less than 10 days. The active principle for inhibition of neem leaves is localized in the organic extractable fraction. Neem supplementation to manure piles on dairies and feedlots, and also to produce fields, could be a novel strategy for on-site pathogen control.
